# Drivers, barriers, and response to care of Australian pregnant women seeking chiropractic care for low back and pelvic girdle pain: a qualitative case study

**DOI:** 10.1186/s12998-023-00516-x

**Published:** 2023-10-03

**Authors:** Maria Bernard-Giglio, Simon D French, Corrie Myburgh, Katie de Luca

**Affiliations:** 1 Department of Chiropractic, Faculty of Medicine, Health and Human Sciences, Macquarie University, Sydney, Australia; 2 Department of Sports Science and Clinical Biomechanics, University of Southern Denmark, Campusvej 55, Odense M, 5230 Denmark; 3 Chiropractic Knowledge Hub, University of Southern Denmark, Campusvej 55, Odense M, 5230 Denmark; 4 Discipline of Chiropractic, School of Health, Medical and Applied Sciences, CQ University, Brisbane, Australia

**Keywords:** Chiropractic, Pregnancy, Low back pain, Pelvic girdle pain, Qualitative case study, Spinal manipulation

## Abstract

**Background:**

Pregnancy-related low back and/or pelvic girdle pain is common, with a prevalence rate of up to 86% in pregnant women. Although 19.5% of Australian pregnant women visit a chiropractor for pelvic girdle pain, little is known about the experience of pregnant women who seek this care. The aim of this study was to describe and explore the experiences of Australian pregnant women who seek chiropractic care for their current pregnancy-related low back and/or pelvic girdle pain.

**Methods:**

A qualitative case study approach with purposive sampling from 27 chiropractic practices was used. A grounded theory approach was informed by a constructivist and interpretivist stance, which provided understanding and meaning to the pregnant women’s experiences. Online unstructured interviews were recorded, transcribed, and anonymised. A thematic analysis was subsequently conducted on the primary data. Codes and major themes were developed with the use of critical self- reflection (memos), survey finding triangulation and respondent validation.

**Results:**

Sixteen potential respondents expressed interest in participating. After eligibility screening and data saturation, nine interviews were undertaken. Four key themes were identified: “Care drivers: what drives care seeking?”, “Care barriers: what barriers are encountered?”, “Chiropractic treatment: what does treatment consist of?” and “Response to care: what response was there to care?”.

**Conclusion:**

Four key themes: care drivers, care barriers, chiropractic treatment, and response to care support an emergent substantive-level theory in women’s care seeking experiences for pregnancy-related back pain and chiropractic care. This theory is that chiropractic care for pregnant women experiencing low back pain and pelvic girdle pain may improve pain and function, while reducing pregnancy-related biopsychosocial concerns. The findings may inform antenatal health providers and the chiropractic profession about pregnant women’s experience seeking chiropractic care as well as directing future research.

**Supplementary Information:**

The online version contains supplementary material available at 10.1186/s12998-023-00516-x.

## Introduction

Pregnancy-related pelvic girdle pain (PPGP) is experienced in the posterior and/or anterior aspects of the pelvis. It may localise in the region of the sacroiliac joints, with or without posterior thigh referral, and in the symphysis pubis [1]. Pregnancy-related low back pain (PLBP) is defined as localised lumbar pain between the 12th rib to the gluteal fold [1]. These pains can occur simultaneously and often referred to as pregnancy-related lumbo-pelvic girdle pain (PLPP) [1]. Pregnancy-related lumbo-pelvic girdle pain (PLPP) is common with a prevalence rate of up to 86% [2]. Women can experience any of these pregnancy-related back pains at any gestational stage, with up to 77% of women reporting pain during the third trimester [3]. For the ease of reading, these three types of back pains will be collapsed and considered “pregnancy-related back pain”.

Pregnant women report a lack of knowledge about pregnancy-related back pain. They also report antenatal health providers normalising the experience of pregnancy-related back pain [2, 4, 5]. While most pregnant women (59-89%) tell their antenatal health providers about their pregnancy-related back pain, up to 53% will receive any treatment [2, 4]. In antenatal pregnancy guidelines, usual care includes exercise, non-rigid belts, medication, reassurance and ergonomic advice for the treatment of pregnancy-related back pain [6, 7].

In Australia, 36.7% of chiropractors reported that they often provide care for pregnant women [8]. Although 19.5% of Australian pregnant women consult a chiropractor [9] specifically for pelvic girdle pain, little is known about these women who seek this care. While a Canadian qualitative study reported a thematic finding of positive responses toward chiropractic treatment in women with pregnancy-related back pain in managing their symptoms [10], it did not examine the experiences of this care seeking pathway. Care seeking experiences have been considered in an Australian study on endometriosis; where endometriosis is a “circuit breaker” - involving pain normalized, daily life disruption and intercession of others - that creates an experience to further seek medical help [11].

Patient experiences are a key component of healthcare quality [12, 13] and patient narratives may provide insight into accessing a model of care for pregnancy-related back pain largely unknown in an Australian setting. Therefore, the aim of this qualitative study was to describe and explore the experiences of Australian pregnant women who seek chiropractic care for their current pregnancy-related back pain.

## Methods

### Design

A qualitative case study method allowed for an exploration of emergent themes in Australian women experiencing pregnancy-related back pain seeking chiropractic care. A grounded theory approach aims to generate theory or investigate emergent substantive theory out of qualitative data [14]. An emergent substantive theory provides a theoretical interpretation or explanation of a phenomenon under study [15]. Grounded theory methods provide a framework of coding for inductive analysis, constant comparative, and iterative analysis of data, and its outcomes [14]. In this study, a grounded theory approach was informed by a constructivist and interpretivist stance. The interpretivist position required the researcher (MG) to grasp the subjective meaning of the chiropractic care seeking experiences of respondents [14]. In a constructivist stance the respondents described the views of their reality, and this enabled the researcher to better understand the respondents’ actions [16].

A grounded theory approach was considered appropriate as the study aimed to draw together the collective insights from individual cases to distil a rich description of the process of this particular instance of care seeking. A multiple case study approach enabled the researcher to explore and compare within and between cases, replicating similar themes expressed as an emergent substantive theory. These themes may provide an emergent substantive theory to be supportive or add to current findings in pregnant women’s care seeking experiences. The consolidated criteria for reporting qualitative research (COREQ) checklist [17] was used to guide reporting of this qualitative study; (See Supplementary Tables 1, Additional File 1).

### Theoretical framework

The theoretical perspective we have drawn upon for this study was based on the work of Manderson et al., [11] who, through a similar qualitative approach, sought to understand the process of care seeking among Australian women with endometriosis [11]. The authors highlighted so-called ‘circuit breakers’ that lead participants to further seek medical advice. Their results included four themes: 1) intercession by significant others who recognised their problems as abnormal; 2) social disruption of their daily lives due to pain; 3) biographical disruptions such as failure to conceive or miscarriage; and 4) self-recognition of likely pathology. Although we made no explicit *a priori* assumptions, our position at the outset of our study was that similar issues have the potential to reflect the interactions and strategies that characterise women attempting to seek care for their current pregnancy-related back pain.

### Sampling strategy

A purposive sampling approach sought to identify pregnant women who met a specific eligibility case bound criterion. An invitation was sent to Australian chiropractic clinics, disseminated via chiropractic association newsletters and social media posts. Chiropractors who responded were provided advertising material to assist the recruitment of pregnant women currently receiving chiropractic care for pregnancy-related back pain. The sample size was not determined a priori; once data saturation [14] was observed, that is, when sampling further cases yielded no further concepts, recruitment was concluded.

### Eligibility case bound criterion

A short phone call between the researcher and respondent established the eligibility and interview date and time. Patient information and consent forms were then sent to the patient. These briefly described the interviewer (MG) as a clinical researcher and that the respondents’ voluntary participation could be withdrawn at any period during the study. A case bound eligibility was established to help manage contextual variables in the unit of analysis, that is, pregnant women with pregnancy-related back pain seeking current chiropractic care. Inclusion criteria were pregnant women: (1) between the ages of 18–35; (2) experiencing a low-risk pregnancy; (3) currently 12–36 weeks gestation; and (4) received at least two or more sessions of chiropractic care for pregnancy-related back pain. Exclusion criteria were pregnant women: (1) of advanced maternal age (> 35 years of age); (2) with previous lumbo-pelvic surgery; (3) with lumbar diseases (known disc herniation or prolapse, spondylolisthesis or known lumbar pathology, including osteoporosis); (4) with inflammatory arthropathies; (5) experiencing a complicated pregnancy including hypertension, diabetes, premature contractions, multifetal pregnancy, placenta previa, known fetal anomalies and any condition deemed as higher maternal risk; and, (6) with insufficient proficiency in English.

#### An unstructured interview

Each interview began with the same question. *“So, you were having low back and/or pelvic girdle pain in this pregnancy and decided to see a chiropractor. Can you tell me about this experience, of you having pregnancy-related low back and/or pelvic girdle pain in pregnancy and seeking a chiropractor?”*

This question provided the women an opportunity to use a reflective stance recounting this experience. The unstructured interview style allowed the interviewee to guide the conversation and course of the narrative [18]. Open-ended questions encouraged a reflective story of the respondents’ experiences, feelings, actions, and thoughts of her pregnancy-related back pain and seeking chiropractic care. For example, in the first interview, R1 began with explaining that she was in an exercise class where the Doula/Yoga teacher recommended chiropractic care for back pain. Further questioning made it clear if she had in fact had back pain prior to her attending exercise sessions. MG *“Were you doing exercise for your pain? Why were you going to yoga?”. R1* “*No I was doing yoga just something to do with other pregnant women, and stretching, but no not because of pain. When pain started in my lower back, I thought Oh I should go to the chiro.”* Further open-ended questions were used to better define the type and location of back pain. The Zoom interview allowed the respondent to describe and demonstrate the site of pain. *MG “Where did you feel your back pain?”*. *R1 “The pain starts right there…”.* R1 stands and demonstrates on her body. *MG “We are talking about the iliac crest, the top of the sacrum area, but does it go into the “cheeks” at all? Or into the legs? In the first pregnancy did it go into the legs at all? R1 “Nupe. I don’t remember ever having leg pain and then just groin pain, which is the pelvic girdle pain, which is just on the front here in the groin* (indicates her front groin right region on her body)”. The respondent was encouraged to describe her pain, location, and its severity.

Emerging concepts and themes were explored in the nine subsequent interviews; this was done by comparing those themes with further emergent concepts and themes that arose from cross-case analysis.

To enhance trustworthiness of data collection [14], interviews did not use a predetermined set of questions. Instead, an unstructured interview style was used to ensure that this approach created sufficient bracketing of the area of interest, in order to allow for cross-case analysis [14]. The interview was piloted with a pregnant woman, whose data was not included in the study.

#### Data collection

In the environment of COVID-19 related social restrictions, one-on-one interviews were performed via an online video platform (Zoom). This allowed an interview setting appropriate to the needs of the respondent and interviewer (MG). Interviews were conducted with audio and optional video recording, using two devices, and transcribed verbatim. Transcripts of interviews, reflective memos, and respondent survey findings were uploaded into the qualitative data analysis software, NVivo-12 Plus program [19]. This software was used to assist organising and managing of the data. All data were decoded and de-identified.

Pregnant women provided sociodemographic and health information and answered the pelvic girdle questionnaire (PGQ) [20] survey prior to the interview. The PGQ responses were used by the interviewer (MG) to clarify if respondents were experiencing symptoms of pregnancy-related back pain. The PGQ responses provided a stimulus upon which respondents could reflect on their current experience of pain and functional abilities prior to, and after, seeking chiropractic care.

Respondents described the severity and location of their pain during the interview. The severity of symptoms and functional ability of these respondents were reported in their written responses in their PGQ. Triangulation of data indicated all the respondents described experiencing moderate or severe pregnancy-related back pain.

### Data analysis

Coding is a strategy used in grounded theory, in which textual data were assigned a descriptive label that allowed the researchers to identify related content across the data. Two independent researchers (MG and CM) initially used inductive coding to reveal common codes and subcodes in the first respondent data. Coding continued until saturation was met at the ninth respondent. The NVivo-12 plus program was used to assist in the organisation of common codes and subcodes. An initial codebook was agreed upon by researchers (MG, CM and KD) using data from the first respondent. This codebook provided a list of common codes, subcodes, and their definitions, to be used as an interview guide. This guide assisted in further questioning to determine the significance of these concepts in the next interviews. An example follows of coding a comment from the first respondent.

*“I was doing, post-natal Yoga and the instructor was also a Doula, so she just recommended going to a Chiropractor, and she recommended this chiropractor, where I am now going, as, she does pregnant women a lot, um and had good recommendations, so I just followed where she recommended me to go and that’s where I ended up.” R1–32 years of age multigravida*.

This section was coded as, “care seeking experiences”. It was defined in the codebook as an action that describes care seeking behaviour. With further interviews and coding, this was further classified into subcodes of: values and recommendations, looking for interventions for pain and chiropractor and patient relationship.

Two independent researchers (MG and CM) subsequently used inductive, deductive, and iterative comparison in the analysis of the third respondent’s data to promote further agreement amongst the coders. A composite codebook was formed with further subcodes and definitions to be used in comparative and iterative analysis among cases. Researchers (MG, CM and KD) further refined the codes and concepts providing a consensus for a composite codebook to guide subsequent interviews [14, 21]. (The composite codebook guide, See Supplementary Tables 2, Additional File 2). This approach provided a conceptual framework for the comparative exploration of common codes, concepts, and themes in cross-case analysis [22]. The researcher (MG) who interviewed respondents used this composite codebook guide to: (1) further data collection; (2) organise coding in the NVivo program; and, (3) analyse common recurring concepts and themes. Iterative analysis, critical self-reflection and condensation of data continued until data saturation.

### Triangulation and respondent validation

Triangulation of data enhances the trustworthiness of the analysis with convergence of information from different data information sources [22]. These sources included: (1) transcribed interviews verbatim; (2) respondent background characteristics; (3) qualitative PGQ responses indicating levels of pain severity and functional abilities before and after chiropractic care; and (4) critical reflective practice (memos). Respondent validation aims to promote trustworthiness, confirmability and credibility [14]. While the process ensures that respondents can recognise their ‘voice’ in the organisation and interpretation of the data and confirm that the researcher has correctly understood these experiences, some qualitative researchers accept that it is not practical for all respondents to be re-interviewed for this confirmation [14]. Time constraints and demands on the study participants who were new mothers were also considered. Validation of final interpretation of the findings was provided by one respondent.

### Reflexivity

Critical reflection was undertaken at each step of the qualitative study. The researcher (MG) used careful self-reflection in this research, aiming to set aside personal views and reactions and listening from the perspective of a researcher. Self-reflexivity continued throughout data analysis with self-checking using memos, regular research team meetings and discussions. As a researcher conducting the interviews, MG is also an experienced chiropractic practitioner in treating women with pregnancy-related back pain. The research team deemed it most appropriate that an unstructured interview method would allow the respondent to guide the interview and allow more follow-up open-ended questions in response to the conversation.

An experienced qualitative researcher (CM) was consulted for the study design, coding, and data analysis. Regular research team meetings provided discussions and iterative comparison of cases with the emergent concepts and major themes.

## Results

### Respondent recruitment

**Fig. 1:**
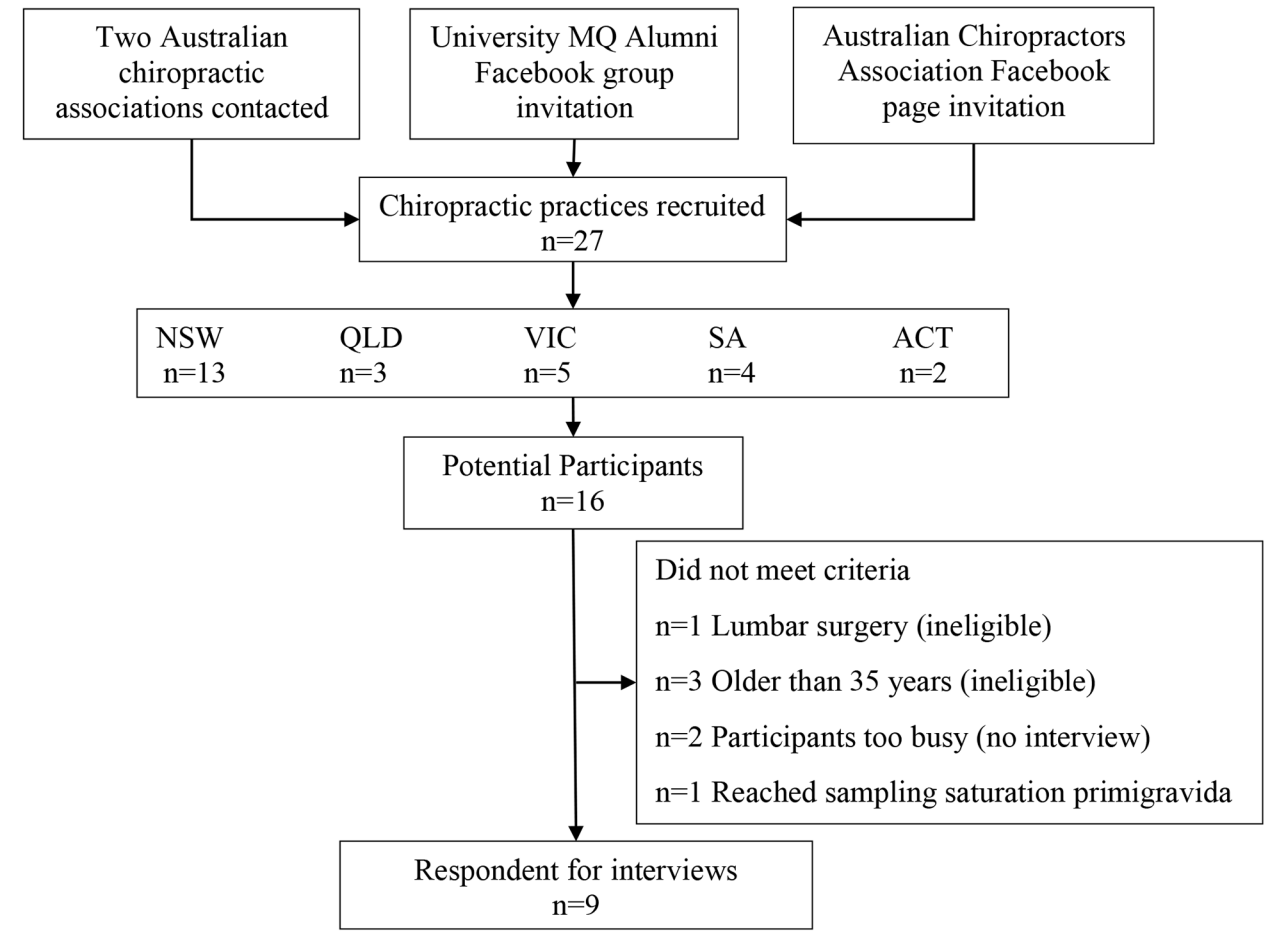
Respondent recruitment flow chart

From August to December 2021, 27 chiropractic clinics responded to advertising, with 16 potential respondents contacting the researcher (MG) to participate. Respondents were screened consecutively and interviewed within a time frame of two or three weeks from first contact. After screening for eligibility, seven respondents who enquired with interest about the study were excluded, due to lumbar surgery (n = 1), older than 35 years (n = 3), too busy to continue with study (n = 2), and because data saturation was reached sampling primigravida (n = 1). This process continued until the study reached data saturation, as agreed upon by the research team (MG, KD, and CM), resulting in nine respondents interviewed (Fig. 1). Interviews between the researcher (MG) and the respondents were undertaken between September 2020 to January 2021. Interviews ranged from 35 to 66 min duration, with an average time of 47 min per interview.

### Characteristics of respondents

Sociodemographic, health and chiropractic care characteristics of the respondents are shown in Table 1.

**Table 1:** Summary of sociodemographic, health and chiropractic care characteristics of respondents

Characteristics	Description	Results
***Sociodemographic***	**Age in years, range (mean)**	19–33 (28.5)
	**Location (n = 9)**	
	New South Wales	3/9
	Victoria	2/9
	Australian Capital Territory	1/9
	South Australia	3/9
	**Education Level (n = 9)**	
	Year 10	1/9
	Diploma/certificate/Degree	8/9
	**Employment (n = 9)**	
	Part-time	4/9
	Full-time	3/9
	Not working	2/9
	**Migrant (n = 9)**	3/9
	**Private Health Insurance (n = 9)**	3/9
***Pregnancy***	**Gravidity (n = 9)**	
	Primigravida (pregnant first time, 5/9)	6/9
	Multigravida (one child)	3/9
	**Previous miscarriages (n = 9)**	2/9
	**Gestation at time of interview in weeks, range (median)**	20–35 (24 weeks)
***Low back and pelvic girdle pain***	**Low back pain prior to pregnancy (n = 9)**	6/9
	**Low back and pelvic girdle pain in previous pregnancy (n = 3)**	3/3
	**Trimester that low back and pelvic girdle pain commenced (n = 9)**	
	Trimester 1	4/9
	Trimester 2	4/9
	Trimester 3	1/9
	**Range (median)**	8–28 weeks (12 weeks)
***Chiropractic care***	**No previous experience of chiropractic care (n = 9)**	3/9
	**Previous chiropractic care in previous pregnancy (n = 3)**	3/3
	**Gestation in weeks at the time respondent started chiropractic care, range (median)**	8–28 (18 weeks)
	**Other than chiropractic care intervention in previous pregnancy care (n = 3)**	3/3

### Themes

**Fig. 2:**
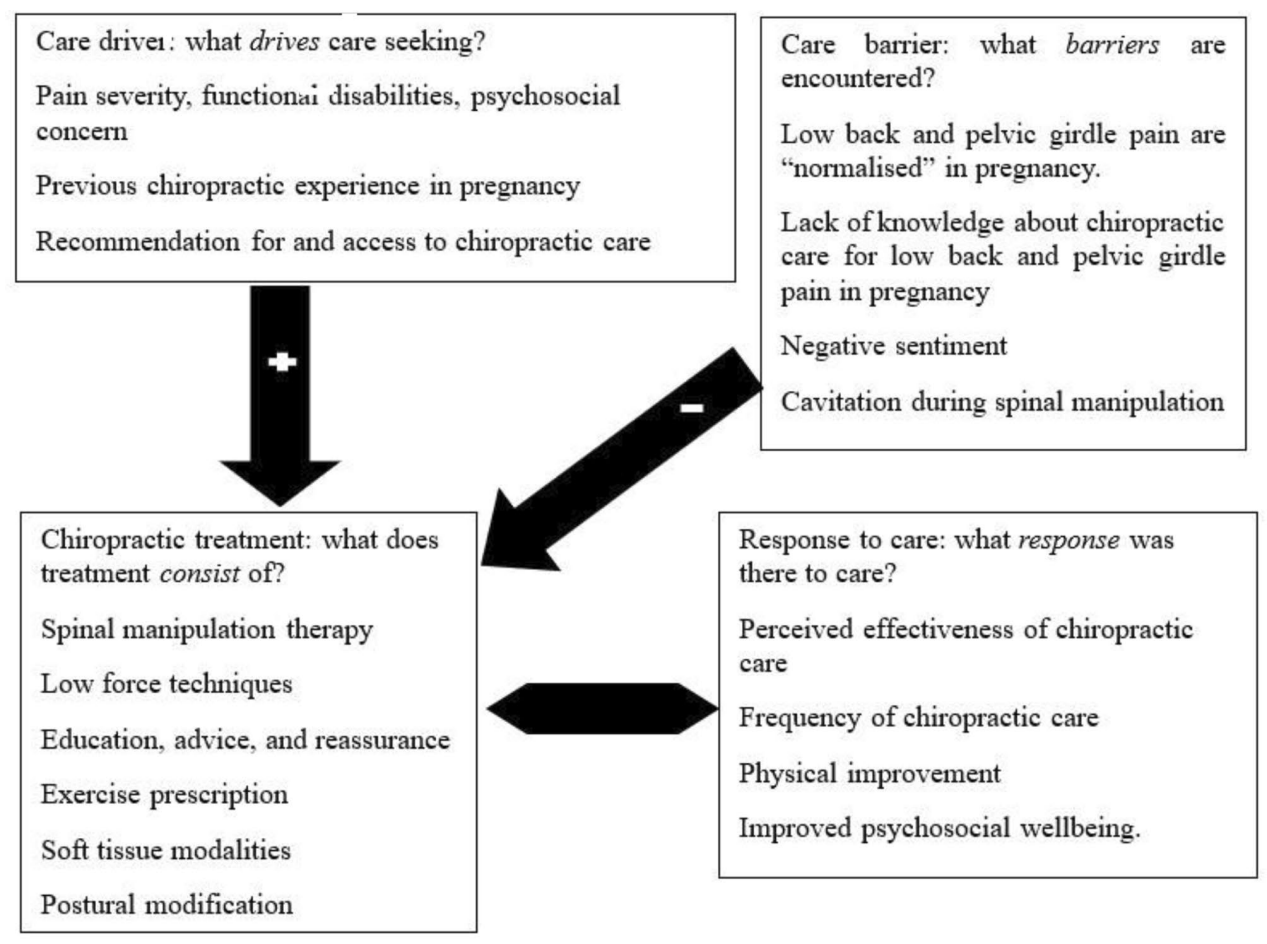
Pathway highlighting drives (+), barriers (‒) to care seeking experiences among respondents

Major themes and subthemes are found in Table 2. Major themes that arose include: “Care drivers: what drives care seeking?”; “Care barriers: what barriers are encountered?”; “Chiropractic treatment: what does treatment consist of?”; and “Response to care: what response was there to care?”. The conceptual framework presents the respondents’ care seeking pathway (Fig. 2). This conceptual framework grounds itself in this unique study including theoretical perspectives based on previous findings by Manderson et al. [11] and thematic analysis by Sadr et al. [10]. These discoveries may formulate a new theoretical framework in care seeking for pregnancy-related back pain and chiropractic care.

**Table 2:** Major themes and sub themes that arose from the description and exploration of Australian pregnant women who sought chiropractic care for their current pregnancy-related low back and pelvic girdle pain

Major themes	Subthemes
**Care drivers**: **What** ***drives*** **care seeking?**	• Pain severity, functional disabilities, psychosocial concern • Previous chiropractic experience in pregnancy • Recommendation for and access to chiropractic care
**Care barriers**: **What** ***barriers*** **are encountered?**	• Low back and pelvic girdle pain are “normalised” in pregnancy • Lack of knowledge about chiropractic care for low back and pelvic girdle pain in pregnancy • Negative sentiment • Cavitation during spinal manipulation
**Chiropractic treatment**: **What does treatment** ***consist*** **of?**	• Spinal manipulation therapy • Low force techniques • Education, advice, and reassurance • Exercise prescription • Soft tissue modalities • Postural modification
**Response to care**: **What** ***response*** **was there to care?**	• Perceived effectiveness of chiropractic care • Frequency of chiropractic care • Physical improvement • Improved psychosocial well being

### Theme one. care drivers: what drives care seeking?

A key respondent perspective that arose included a decision-making process that motivated respondents to seek chiropractic care. Increasing experiences of severe pregnancy-related back pain impacted their function and quality of life which ultimately increased their concerns and/or anxiety in their pregnancy and impending labour/birth. This concern motivated respondents who wanted to take control of their experience of pain.

*“It was beginning to impact my work, the impact of just being able to move, which is really, important for me, I am not someone who just wanted to put my feet up and you know, when it was that point that, I was not even enjoying even able to walk, I thought something has to change here!” R2-29 years of age primigravida*.

Respondents also stated that they were looking for other solutions to their pain than the usual advice of mild analgesics, ergonomic and exercise advice.

*“Yeh they* (Hospital emergency unit - MG) *checked me out, they checked the baby out, it was all good, Um I was happy about that, but I was like, why am I still in so much pain?… “Its maybe just pelvic girdle pain, and they did say it was painful, but they kinda like, I don’t know, the way they talked about it, sort of, downplayed, the way I was actually feeling. “You know just go home have some Panadol”, …and I was like, is there anything else, I can do? And they were like, oh like a heat pack or cold pack but only for 20 min. .And um on your back not on your tummy, obviously. I did do that, and the heat pack really helped, when it was really warm, but then when I had to take it off, it was like just back to horrible.” R3 − 19 years of age primigravida*.

Multigravida respondents shared their previous experiences with chiropractic care for pregnancy-related back pain. Previous chiropractic care was a key driver for respondents to return to care for pregnancy-related back pain in their subsequent pregnancy as they had experienced pain relief and improved function.

*“The reason that I was not that bad was because I acted quite quickly when I felt the pain, and I did that pretty quickly with pregnancy one as well, so I was not going to sit around and be in pain when I knew I could do something about it,” R1–32 years of age multigravida*.

Another key driver to return to chiropractic care for pregnancy-related back pain was a previous positive birthing experience after chiropractic care. One multigravida respondent attributed her positive birthing experience to the regularity and adherence to her chiropractic treatment leading up to the birth.

*“So, I do believe, it was because of my chiro, he* (the baby - MG) *did not get distressed, … And I do believe that I didn’t have to have interference because, maybe I went to the chiro leading up to my birth, I was going maybe twice a week for 3 weeks, just before my due date.” R1- 32 years of age multigravida*.

A key driver for chiropractic care was the advice received from trusted recommendations and “research” from social media and google reviews. Care for their pregnancy-related back pain during pregnancy needed to be accessible, and appointments needed to be convenient to their busy lifestyle.

“*I just trusted what this woman* (doula/yoga teacher - MG) *was saying was valid and I thought I would try it out for myself. I am not that kind of person who would ever just go try chiro just by looking up goggle and try to find someone, I always try find someone with a recommendation. Yep, so the fact that she recommended someone, who was well known in* (local area - MG), *close to me, who, specialized with pregnant women, I was like, yep, that’s where I am going…” R1- 32 years of age multigravida*.

### Theme two. care barriers: what barriers are encountered?

Respondents revealed a lack of knowledge as a barrier; not only their own lack of knowledge on pregnancy-related back pain and the knowledge/benefits of chiropractic care while pregnant but also the lack of knowledge of their antenatal health provider on pregnancy-related back pain and chiropractic care. While all respondents understood and accepted the body changes in pregnancy, they were not expecting the severity of the pregnancy-related back pain they experienced.

*“I was only 8 weeks pregnant, I knew from the dating scans, I knew I was pregnant, so for the last couple of weeks I had been quite sore, I was really struggling a bit, so I could even like not lie on my sofa or sit and watch television at night. This is really uncomfortable, and it was my hip, my right hip and into my leg, so I was like, was it sciatica, …you know. Who do I see about that?” R2- 29 years of age primigravida*.

Most respondents revealed they encountered negative sentiments about the safety of chiropractic during pregnancy and experienced doubt with respect to the chiropractor’s role in treating pregnant women from family members, friends, and other antenatal health professionals. These respondents experienced misconceptions around chiropractic care, which posed as a barrier to seek out chiropractic care for pregnancy-related back pain.

*“I didn’t think it was allowed.* (To consult a chiropractor - MG) *Just because it was, you know, um to do with your central nervous system, you know how they say you can’t even get massages and certain pressure points, so I thought chiro was a “no no”.” R7 -31 years of age primigravida*.

*“She* (the Pilate’s instructor - MG) *said, “Ok, are you going to see a physio about that* (pregnancy-related back pain - MG)*?”, and I said, “I have seen a chiro.” Her face was like, “chiro, with all that popping, in pregnancy, with a bub in there, … umm I really would be seeing a physio.” R2 -29 years of age primigravida*.

The final care barrier was the affronting sound of cavitation during spinal manipulation. The sound was considered a source of negative sentiment for family and peers without first-hand experiences or knowledge of chiropractic. Respondents found it difficult to persuade other pregnant women, unfamiliar with chiropractic care, that they did indeed experience safe and effective care during their pregnancy.

*“Yes, that they don’t understand how a chiropractor could help in pregnancy or they are not hurting you… because all they know is like, is that they crack you or something, like its bones cracking, so people I do talk to, so know, now that I know what they do, I get really frustrated. It’s hard for me to explain what really happens, like it’s no, people don’t really get it.” R5–30 years of age primigravida*.

### Theme three. chiropractic treatment: what does treatment consist of?

Respondents reported receiving similar modalities of care from their individual chiropractors including spinal manipulative therapy (SMT), low force techniques (for example, using an Activator instrument), education, advice and reassurance, exercise prescription, soft tissue modalities, and postural modification. Respondents reported that low force techniques were considered gentle and a different approach by the chiropractor.

*“They* (different chiropractors in pregnancies - MG*) have been quite different, actually, interestingly, with different techniques, Well so the chiropractor I had seen in my first pregnancy, I guess, I don’t know what you call them? The tool? Whatever tool you use.*

“Activator? the clicker gun?” - MG.

*Yeh. The clicker thing, it looks completely bizarre, yeh, it clicks.*

*The chiropractor this time round doesn’t even use that, she uses more manual techniques”.*

*R9–33 years of age multigravida*

Most respondents were not familiar with the language of manual therapy. They used “adjustment” instead of SMT, and commonly used the word “crack” to explain the sound of cavitation and the action of receiving SMT.

*“She is putting 2 little foam things under my hip to kind of align them and she is massaging out my back a lot. Last time I went, she cracked my back.” R1–32 years of age multigravida*.

Postural advice was given by chiropractors for home duties and work, as were instructions on self-management techniques such as stretching, the use of devices including a pelvic girdle belt, a foam roller and use of a pregnancy pillow for comfort, and ice packs for pain relief. Respondents recalled that initially during care, stretches and low force exercise, such as walking, were prescribed. As pain decreased, respondents were asked to increase intensity. This may have included adding strengthening exercises, such as yoga or light weights, where comfortable.

*“I just remembering trying, obviously with that tennis ball, and rolling on it and really arghh… over the hip and then I could barely walk. By the Monday when I saw the Chiro, he was like, whoo, no I recommend ice for that, so regularly after that I was putting ice on my .and it just helped. To the point that, you know for the first probably 5–6 weeks, I was using icepacks for a couple of weeks, and doing the exercises and now I literally, I am now at the point, I just have no pain.” R2 -29 years of age primigravida*.

*“The chiro was like, maybe we shouldn’t be doing anything one legged because it was kinda reverting back to the pain in my hip and this inflammation. I am not doing curtsy lunges, anymore, I won’t do that anymore. Again, in fact any time I do a curtsy lunge, I never feel great, because that is purposely putting your whole pelvis off. But a lunge, a vertical kinda of lunge, with weights was feeling absolutely fine. So, I am actually 23 weeks pregnant and I am actually able to do that, better than what I was 9–10 weeks pregnant.” R2–29 years of age primigravida*.

### Theme four. response to care: what response was there to care?

All respondents described a reduction in their levels of pain and improvements in their ability to continue with occupational and home duties. This response to care varied between respondents, as was the time to recovery from their episode of pregnancy-related back pain. For some primigravida, they were surprised by how quickly chiropractic care gave them pain relief, while others felt relief was short-lived.

*“Initially, I was quite shocked that he got me up and he did an adjustment* (spinal manipulation - MG) *… and I said, “is that it?” well that’s not going to work … Yeh, well in the next few days, I remember being like, Oh, this is feeling a bit, a bit better.” R2 -29 years of age primigravida*.

*“I kinda thought I was a bit worried, that it would hurt. I was a little bit concerned that, movement and jolting, that would hurt. But it didn’t. I was really pleasantly surprised.” R6–29 years of age primigravida*.

*“She basically did just one adjustment, and from then I saw her weekly for about a month, now I ‘ve spaced it out to 2 weeks, because I don’t feel that I need to see her, as much,” R7–31 years of age primigravida*.

Some primigravida women were unclear about how long they required treatment for, or if ongoing management would help. Some primigravida managed their pregnancy-related back pain with regular, frequent chiropractic care.

*“It felt like it would feel good for a few days after, then the disability, would come back again. It lasted to me, it felt like it lasted forever. It did probably, it in a month maybe, um, that’s when my leg wasn’t giving way, I could walk, I could stand, I could actually move, and not feel anything,” R5 -30 years of age primigravida*.

Recovery from pregnancy-related back pain and disability also improved psychosocial well-being, including better mental health and reduced stress and anxiety.

*“Then it was after I went and got my first adjustment done, when I went home, I was actually able to eat some crackers, and have a bit of water, so I was happy about that.” R3 -19 years of age primigravida*.

*“I’m still in disbelief how it actually works, because I’m feeling like I’m in there for several minutes, and you know it’s not the cheapest thing in the world, but I spoke to hubby about it, and he was like, the difference in you love, is that it is absolutely worth its weight in gold!” R2–29 years of age primigravida*.

## Discussion

This qualitative case study is the first systematic, empirical exploration of the experiences of pregnant women seeking chiropractic care for their pregnancy-related back pain in an Australian context. Key themes that arose include care drivers, care barriers, chiropractic treatment, and response to care. Based on our analysis of key thematic issues observed amongst our respondent group, the present study contributes substantively to our understanding of this uniquely female healthcare problem. The study has highlighted the difficulties around care seeking where pain and discomfort are normalised, and the emergence of a potentially valuable, yet under-utilised care pathway for pregnancy-related back pain. The themes identified in this study provide substantial alignment with the ‘circuit breaker’ model developed by Manderson et al. [11]. Specifically, we observed evidence of all four circuit breakers, primarily under our care driver and barrier to care themes.

A care driver was this change in pregnant women’s perception of their health condition, i.e., from seeing pain and functional disabilities as “normal”, common and expected [4, 23]. These women were seeking a path to break their cycle of “normalized” pain and functional disabilities. In this study, respondents were driven by psychosocial concern and recognition of pain as abnormal, which led them to seeking for medical care. Care drivers were recommendations for, and access to, chiropractic care by the intercession of significant others and web-based sources. A reduced capacity to perform activities of daily living created significant social disruption amongst our respondents, which in turn drove them to seek care from previously unexplored sources. Psychosocial concern regarding a difficult or complicated delivery and previous positive experiences with chiropractic treatment, were identified as care drivers that speak to the notion of precautions against the circuit breaker of biographical disruption.

These care driver findings of recognising that pain was not “normal”, facilitated a pathway beyond the boundaries of mainstream medicine. It seems that there may be a category of female reproductive system problems that have similar factors shaping attitudes to care seeking, care seeking behaviours and care provision. With regards to the latter (and in relation to themes two, three and four) our results are suggestive of a care pathway that support an emerging substantive-level theory that chiropractic care for pregnant woman experiencing pregnancy-related back pain may improve their pain, function, and pregnancy-related biopsychosocial concerns.

Respondents lacked knowledge about pregnancy-related back pain [4, 24] which formed a barrier in seeking care (Theme two). The intercession of significant individuals may inform pregnant women [25], but attitudes of negative sentiments were also likely to turn away pregnant women from seeking chiropractic care for pregnancy-related back pain. In a systematic review on adverse reactions and chiropractic care in pregnancy-related back pain, the authors concluded that adverse events are rare in these populations with a handful of minor and transient adverse musculoskeletal events following lumbar spine manipulation [26]. International best practice guidelines on the treatment of pregnancy-related back pain suggest a trial of manual care, as long as treatment does not increase pregnant women’s symptoms [27, 28].

The third theme that emerged from our study, “Chiropractic treatment: what does treatment consist of?” complements themes from Sadr et al. [10] who identified five themes including: treatment and effectiveness; chiropractor-patient communication; pregnant patient presentation and chiropractic approach to pregnancy care; safety considerations; and self-care. Chiropractic care received by the women in this current study is similar to previous research [10, 29] and to best practice guideline recommendations [27].

The fourth theme that emerged from our study, “Response to care: what response was there to care?” demonstrated that by possibly reducing pregnancy-related back pain and psychosocial concerns, pregnant women may also have reduced risks associated with poor maternal health outcomes [30]. These respondents are part of a global concern where the prevalence of unresolved pregnancy-related back pain in women, for up to 12 years post-partum, is 21% [31]. With the detrimental impact that pregnancy-related back pain has on activities of daily living [2, 24, 32–34], anxiety and depression [30, 35], post-partum pain [36, 37], and the financial disadvantage in pregnant women [38, 39] includes the loss of income from sick leave [30], chiropractic as a safe and effective non-pharmaceutical treatment option is perhaps a key reason for seeking care [10, 29].

### Strengths, limitations, and future research

This study sought to understand the experiences of seeking chiropractic care for pregnancy-related back pain and develop an explanatory framework, or “theory” using grounded theory methods. Pregnant women were invited to tell us about care seeking experiences, and a process of data collection and analysis shaped the collection of concepts and themes. This study provided substantive theories to promote further investigation of pregnancy-related back pain and chiropractic care.

Our study was not just descriptive of experiences. Data has been treated as potential indicators of concepts, and these indicators were constantly compared to see which concepts they fit best with. These results were looking towards making sense of what the experience means; substantive theories, which require further study. Nevertheless, limitations include that other phenomenological framework with qualitative content analysis, such as interpretative phenomenological analysis (IPA), may have been viewed as better positioned to understand the life experiences of pregnant women.

In regard to recruiting advanced aged gravidas (> 35 years) in future studies may provide insight of mature women and/or with larger families. Early gestational pregnancy-related back pain experiences were captured, but the exclusion criterion failed to allow advanced gestational age experiences (> 36 weeks of pregnancy) in seeking care. Furthermore, women with existing lumbar disease were not recruited, and our sample failed to include low socio-economic women and women without healthcare insurance. However, these women are less likely to seek help for their pregnancy-related back pain symptoms [9]. Finally, a lack of contrasting experiences in the theme “Response to care: what response was there to care?” is a limitation which reduces the generalisability of this study.

It is suggested that future studies provide clear and standardised definitions for the subgroups of pregnancy-related back pain including PLBP, PPGP or PLPP [40, 41]. Standardised definitions of pregnancy-related back pain may help in understanding the aetiology, progression, and measuring the effectiveness of chiropractic care in each subgroup. Future studies should explore force parameters of spinal manipulative therapy in pregnant women. Future studies should incorporate appropriate outcome measures that assess pregnant women’s pain, functional ability and health beliefs and outcomes of care [42, 43]. Importantly, future studies should determine the effectiveness of chiropractic care and report adverse events.

## Conclusion

This qualitative study provided key themes that arose from pregnant women currently experiencing pregnancy-related back pain and seeking chiropractic care in an Australian setting. The four key themes identified were care drivers, care barriers, chiropractic treatment, and response to care. The themes identified may help the integration of chiropractic care in formal antenatal care where it is not well understood how chiropractic care may improve pregnancy-related back pain. These themes support an emergent theory for future trials to determine the effectiveness of chiropractic care for pregnant woman experiencing pregnancy-related back pain. Due to the qualitative study design, findings have only naturalistic generalisability and general transferability. However, the findings may inform antenatal health providers and the chiropractic profession about pregnant women’s experience seeking chiropractic care.

### Electronic supplementary material

Below is the link to the electronic supplementary material.

Supplementary Material 1

Supplementary Material 2

## Data Availability

The datasets used and/or analysed during the current study are available from the corresponding author on reasonable request.

## List of Abbreviations

CAM: Complementary alternative therapists
COREQ: Consolidated criteria for reporting qualitative research
CM: Corrie Myburgh
KD: Katie de Luca
MG: Maria Bernard-Giglio
LBP: Low back pain
LPGP: Lumbo-pelvic girdle pain
PGP: Pelvic girdle pain
PGQ: Pelvic Girdle Questionnaire
SF: Simon French
SMT: Spinal manipulative therapy
VoIP: Voice over Internet Protocol
R1: Respondent one
R2: Respondent two
R3: Respondent three
R4: Respondent four
R5: Respondent five
R6: Respondents six
R7: Respondent seven
R8: Respondent eight
R9: Respondent nine
