# Supplementary material for: Drivers, barriers, and response to care of Australian pregnant women seeking chiropractic care for low back and pelvic girdle pain: a qualitative case study
Source: Chiropr Man Therap. 2023 Oct 3;31:43. doi: 10.1186/s12998-023-00516-x (PMC10546639; doi:10.1186/s12998-023-00516-x)
Supplement: Supplementary file 2 — Supplementary Material 2 [file 12998_2023_516_MOESM2_ESM.pdf]

Supplementary Table 2, Additional File 2

Composite codebook and definitions with subcodes

Code Name	Comment/Definition	Subcode Names
**Accessibility**	Code that highlights factors that may determine accessibility to chiropractic care (the quality of being easy to use)	Childcare factor
		Location of chiropractic clinic
		Managing time
		Financial accessibility
**Care barrier**	Code highlights factors that will dissuade the seeking of chiropractic care	Self-funded costs
		Negative sentiments
		Lack of knowledge on LB/PGP in pregnancy
		Lack of knowledge on LB/PGP and chiro care
		The "sounds" joint cavitation
		Adverse reaction
**Care driver**	Code the indicates factors that will promote the seeking of chiropractic care	Personal positive experience
		Attributes of the chiropractor
		Severity of pain and functional disabilities
		History of previous LB/PGP in pregnancy and chiropractic care
		Locus of control
**Care seeking experiences**	Code that describes care seeking behaviour exhibited	Values and recommendations
		Looks for other interventions for pain
		DR of chiropractic and patient relationship
**LB/PGP expert guidance**	Code highlights the use of those with "expertise" in LB/PGP to influence respondent seeking behaviour	Antenatal carer, GP share care
		Alternative antenatal carer
**Explanatory framework-patient**		The nature of LBP-related problems
		Understanding LB/PGP in pregnancy

**Healthcare beliefs**	Code describes beliefs that influence health care-seeking behaviour	
**Interprofessional practice or collaborators**	Code indicates collaborative practice between health care providers	
**Chiropractic treatment**	Code describes the treatment and adjunctive care by chiropractor	Spinal manipulation treatment (SMT)
		Massage
		Devices or modification of techniques
		Exercise
		Postural modification
		Education and reassurance
**Pain location**	Pain location defines LBP or PGP or LB/PGP	
**Respondent profile**	Code describes respondent characteristics	
**Response to care**	Code describes respondent status in process of dealing with their LB/PGP	Respondent recovery status
		Duration of perceived effectiveness
		Perceived effectiveness -global satisfaction
		Perceived effectiveness outcomes of care
**Locus of control**	Code that describes the belief of respondent establishing control over their own health	
**Support**	Code highlights the role of support associated with seeking care	Spousal or partner
		Family/peers
		Antenatal carers
